# Synchronous unilateral basal cell adenoma of the parotid gland: A case report

**DOI:** 10.3892/ol.2014.2334

**Published:** 2014-07-10

**Authors:** JIN KUANG, QIAN RAO, HAO ZHANG, ZHIGANG CHENG

**Affiliations:** 1 Department of Oral and Maxillofacial Surgery, Wuhan Central Hospital, Wuhan, Hubei 430014, P.R. China; 2 Department of Dentistry, Wuhan Children’s Hospital, Wuhan, Hubei 430015, P.R. China

**Keywords:** basal cell adenoma, synchronous, parotid gland

## Abstract

The current study reports the case of a 68-year-old male who presented with a 4-month history of a painless slow-growing mass in the left parotid region. Magnetic resonance imaging revealed two independent, round lesions in the superficial and deep lobes of the parotid gland on the left side, respectively. A total parotidectomy was performed and basal cell adenomas (BCAs) were identified by histopathological examination. At the 6-month follow-up examination, no sign of recurrence was found. This study describes the clinical features of a rare case of synchronous unilateral BCA in the parotid gland and also provides a review of the literature.

## Introduction

Basal cell adenoma (BCA) was first described in the salivary gland in a study by Kleinsasser and Klein in 1967 (1). This tumor represents 1–2% of all salivary gland tumors, and the majority of these are found in the parotid gland. In 1991, BCA was recognized as an independent entity in the second edition of the Salivary Gland Tumors Classification by the World Health Organization (2). Histologically, this tumor is composed of basaloid cells delineated from the stroma by the basement membrane. There are four characteristic patterns of BCA; solid, trabecular, tubular and membranous. The membranous subtype exhibiting high recurrence rates. Histological and immunohistochemical staining are used for diagnosis, while surgical resection with a cuff of normal salivary tissue is the main treatment. The current study reports a rare case of synchronous BCA of the left parotid gland in a 68-year-old male. In addition, the clinical features of the condition are described and a review of the literature is presented. This study was approved by the Ethics Committee of Wuhan Central Hospital (Wuhan, China) and was performed according to the Declaration of Helsinki. Patient provided written informed consent.

## Case report

In November 2012, a 68-year-old male presented to the Wuhan Union Hospital (Wuhan, China) with a mass in the left infra-auricular area was referred to the Department of Oral and Maxillofacial Surgery at Wuhan Central Hospital (Wuhan, China) in March 2013. Four months prior to admittance, an ultrasound examination at Wuhan Union Hospital identified a homogeneous tumor in the left parotid region. A fine-needle aspiration biopsy extracted brown liquid indicative of a cyst of the parotid gland.

Upon physical examination, a round, 1.5×1.5-cm, movable, tender and painless mass was palpable on the superior portion of the parotid gland of the left side. The tumor was not attached to the skin, and no facial palsy or regional lymphadenopathy was observed.

**Figure 1:**
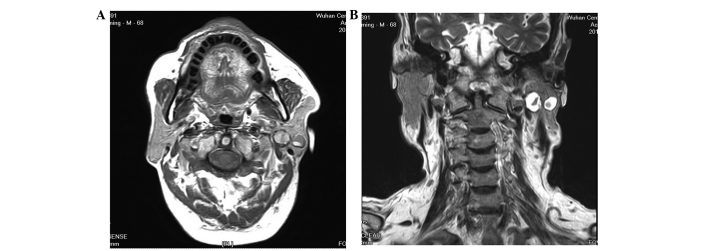
(A) Axial T1-weighted magnetic resonance imaging showing two independent masses in the superficial and deep lobes of the parotid gland on the left side. The two tumors were well-marginated. (B) Coronal T2-weighted magnetic resonance imaging showing moderate enhancement in the peripheral component and signal hypointensity in the central component of the two tumors.

**Figure 2:**
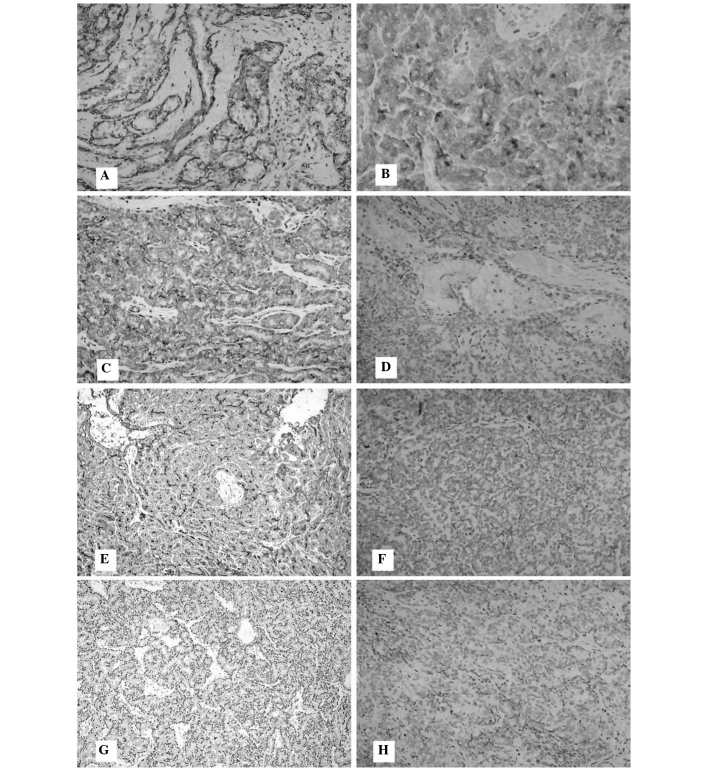
Immunohistochemical staining. The tumor was positively stained for (A) smooth muscle antigen, (B) cluster of differentiation 117, (C) calponin, (E) carcinoembryonic antigen and (G) p63. The tumor was negative for (D) Ki-67, (F) epithelial membrane antigen and (H) p53 (magnification, ×20).

Magnetic resonance imaging (MRI) was performed and showed two independent masses in the superficial and deep lobes of the parotid gland on the left side, respectively (Figs. 1 and 2). The tumors were well-marginated, with peripheral solid and central cystic components. The superficial tumor measured 12 mm in diameter, whereas the deeper tumor measured 15 mm in diameter. From these results, the initial diagnosis was of synchronous unilateral tumors, similar to Warthin’s tumors.

The MRI features on the T1-weighted images revealed differences in the composition of the tumors. The solid component of the superior tumor returned a hypointense signal, higher than that of muscle, but lower than the surrounding parotid tissue. For the deep mass, however, the solid component exhibited slight hyperintensity compared with the superior tumor, and isointensity compared with the surrounding parotid tissue. Compared with the central component of the two masses, the superior tumor exhibited moderate enhancement and the deep tumor was slightly hypointense. On T2-weighted images, moderate enhancement was observed in the peripheral component and hypointensity in the central component.

**Figure 3:**
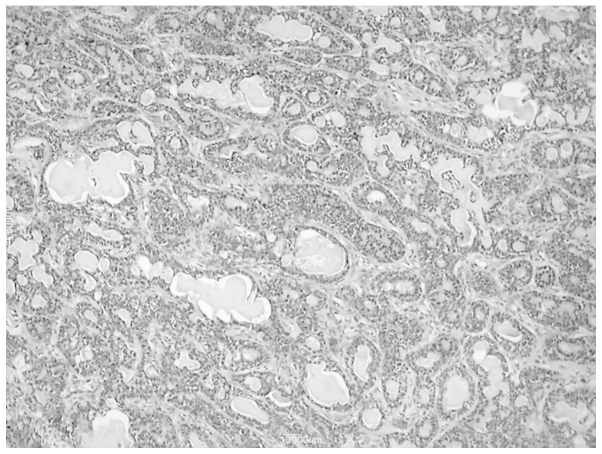
Hematoxilin and eosin staining (magnification, ×20) showing the epithelial tumor separated by stromal tissue, and basaloid cells dispersed in an external stockade pattern.

A total parotidectomy was performed, which included resection of the two tumors and preservation of the facial nerve. Hispathological examination and immunohistochemical study demonstrated that the tumors were BCAs (Figs. 3 and 4). After 6 months of follow-up, no sign of recurrence was found and the facial nerve function had recovered well.

## Discussion

Synchronous unilateral or bilateral multifocal tumors of the salivary glands rarely occur, representing <1% of major salivary gland tumors (3). Adenolymphoma is the most common type of multifocal tumor (4,5). BCA is an uncommon benign neoplasm, accounting for ~2% of tumors in the salivary glands, and with the majority found in the parotid gland.

The occurrence of synchronous bilateral BCAs of the parotid gland is also rare, with only four previously reported cases (6–9). Synchronous unilateral BCA in the parotid gland is extremely rare, and has only been reported once by Kuratomi *et al* (10) in 2006. This study described the case of an elderly female with two simultaneous BCAs as recurrent tumors of pleomorphic adenoma (PA) of the left parotid gland.

Clinical palpation is poor at detecting multifocal ipsilateral tumors, particularly for those tumors that occur in the deep portion. The use of imaging techniques is necessary pre-operatively. Studies on MRI and computed tomography (CT) for the assessment of BCA are few in number. Kiyosue *et al* (11) first reported the MRI findings of BCA of the parotid gland. In the study, BCA was well circumscribed with a rounded shape. The solid section of the tumor exhibited a lower intensity signal than that of the surrounding parotid tissue on T1- and T2-weighted images. Ethunandan *et al* (12), however, found that imaging investigations were able to diagnose only 23% of ipsilateral multiple tumors, while another 56% of tumors were noted by palpation during the surgery, and therefore suggested the use of intra-operative palpation to evaluate the presence and location of multiple tumors.

Differential diagnoses for BCA of the parotid gland include PA and Warthin’s tumors. A mass with lobulated contours favors the diagnosis of a PA, while cyst formation is more common in Warthin’s tumors (13,14). Kuratomi *et al* (10) found that epithelial tumor cells of PA may form BCA through certain differentiation mechanisms. This was a result of the authors identifying that basal cells of the epithelium of PA possess reserve cell functions, through epithelial-mesenchymal transdifferentiation, forming the predominant basaloid cell population of BCA. Chawla *et al* (15) described the CT appearance of 14 cases of BCA of the parotid gland and found the presence of linear bands or stellate-shaped non-enhanced areas may be a specific imaging feature of the tumor.

Histologically, BCA is composed of basaloid cells that are sharply delineated from the stroma by the basement membrane. The absence of a chondromyxoid stroma may be used to distinguish the tumors from PA (16). There are four characteristic patterns of BCA: Solid, trabecular, tubular and membranous. The membranous subtype forms 10% of BCAs, and is often non-encapsulated, multicentric and multilobular, with a post-resection recurrence rate of up to 25% (16). The other subtypes, however, have low recurrence rates due to the absence of pseudopodia (17).

The present study encountered a rare case of synchronous BCA of the left parotid gland. Local excision or extracapsular dissection is not suitable for multifocal ipsilateral or non-encapsulated tumors, therefore, the present case underwent a total parotidectomy.
